# Insulin-Mimic Components in *Acer truncatum* Leaves: Bio-Guided Isolation, Annual Variance Profiling and Regulating Pathway Investigated by Omics

**DOI:** 10.3390/ph14070662

**Published:** 2021-07-11

**Authors:** Xiao-Yue Zhang, Yi-Han Liu, Da-Zhi Liu, Jia-Yang Xu, Qiang Zhang

**Affiliations:** Shaanxi Key Laboratory of Natural Products & Chemical Biology, College of Chemistry & Pharmacy, Northwest A&F University, Yangling 712100, China; xiaoyue.zhang@nwafu.edu.cn (X.-Y.Z.); yhliu@nwafu.edu.cn (Y.-H.L.); Dazhi.Liu@nwafu.edu.cn (D.-Z.L.); jiayang.xu@nwafu.edu.cn (J.-Y.X.)

**Keywords:** hypoglycemic effect, glucose uptake promotion, chemical component, multi-omics, toll-like receptors

## Abstract

Insulin mimic can promote transporting glucose to muscle tissue and accelerate glucose consumption. It is commonly occurring in many functional foods or traditional medicines. Anti-diabetes molecules from food sources are highly safe and suitable for long-term use to prevent early diabetes. The leaves of *Acer truncatum* was found glucose uptake promotion in our phenotypic screening. However, its bioactive components and mechanism are still unclear. We collected leaves from trees of different ages (2, 3, 4, 7 and 11 years old) and profiled the ingredients by LC-MS/MS. The essential active component (myricitrin) was acquired following bio-guide on a whole organism Zebrafish (*Danio rerio*). Its content in the leaves was not affected by tree ages. Therefore, myricitrin can serve as a quality mark for functional foods derived from *A. truncatum* leaves. The transcriptomic and metabolomic analysis in Zebrafish explored the differentially expressed genes and metabolites. Based on joint-pathway enrichment and qRT-PCR verification, the critical bioactive component myricitrin was found to affect toll-like receptors signaling pathways to regulate glucose uptake. Our findings disclosed a bioactive marker (myricitrin) in *A. truncatum* leaves and explored its regulation mechanism, which rationalized the anti-diabetes function of the herbal food.

## 1. Introduction

Diabetes is a chronic metabolic disease that is hard to eradicate and accompanies severe complications, among which Diabetes mellitus type 2 (T2DM) patients are mainly distributed in adults 20–79 years old [1]. The number of diabetes is expected to be double in 2045 [1]. Persons living with diabetes die mostly from related complications instead of diabetes itself [2,3]. T2DM was considered arising primarily from unhealthy diets, such as high-calorie food. However, low carbohydrate diets and fresh fruits have beneficial effects on T2DM [4,5,6]. Thus, diet therapy might be used to prevent early diabetes development. For chronic diseases, prevention is more practical than treatment when suffering from hyperglycemia disorder. Insulin is an essential regulator for maintaining glucose homeostasis in vivo, promoting glucose uptake into muscle and adipose cells to consume extra glucose. Those active small molecules that can improve glucose uptake are also called insulin-mimic. Many insulins mimics were found in foods or herbs, such as coumarin [7] and berberine [8].

Exploring active natural products in traditional medicines faces many challenges. Natural products have not only complex structures but also have multi-target mechanisms. It is difficult to grab all the chemical features and activity mechanisms by traditional isolation and phenotypic screening due to the sample quantity limitation. In recent years, LC-MS/MS and gene sequencing technology have made significant progress. LC-MS/MS profiling associated with database annotation can better interpret the diversity of natural products and conveniently track the dynamic changes in natural sources [9]. Moreover, mRNA sequence and in vivo metabonomics analysis can mine multi-target regulatory network information for natural products in limited quantity [10,11]. Recently, omics analysis has become an attractive strategy in system pharmacology and bioinformatics, which can investigate the effects of drugs or diseases on the whole genome, proteome and metabolome [12,13]. Compared with the traditional phenotypic verification approach, it is easier to find mainly affected pathway by diseases or drugs which can be used for drug repurposing and optimizing treatment. Usually, multi-omics mines differentially expressed transcriptome and metabolome among different biological treatment groups, including blank control and model control groups. In combination with KEGG or other databases, data mining in omics can enrich pathways regulated by natural products [14]. This approach provides a new strategy for interpreting regulation mechanisms for natural products or natural medicines.

*Acer truncatum* is an attractive resource plant for multi-values, such as edible, medicinal, ornamental, ecological and industrial usages [15]. The leaves of *Acer truncatum* can be used for tea or folk medicines [16,17]. In our phenotypic screening of food herbals, we found that the leaves of *A. truncatum* showed prominent promotion of glucose uptake. The leaves, branches, seeds and flowers of *A. truncatum* contain many phenolic substances, such as flavonoids, tannins and lignans [15,18,19], most of which showed potential antioxidant activity [16]. However, the hypoglycemic effect components in *A. truncatum* leaves are still unclear. Herein, we applied bio-guided separation and multi-omics tools to explore the bioactive ingredients, component features in *A. truncatum* leaves, and the regulation mechanism of glucose uptake.

## 2. Results and Discussion

### 2.1. Bioactivity-Guided Isolation

The component features in *A. truncatum* leaves had already been profiled on the basis of LC-MS/MS [15,18]. However, essential components with promoting glucose uptake are still unclear. Therefore, we carried out chemical separation and structural identification based on NMR and MS spectra to obtain active chemical entities to verify the biofunctions and explored the regulation mechanisms.

Bioactivity-guided separation is an effective strategy to find valuable components from natural sources. It can also rationalize the biofunction of traditional medicines or plant herbs. Due to the minimal quantity of fractions in separation, only a tiny enough bioassay model can adapt to the bio-guided separation. Furthermore, the regulation of glucose homeostasis is a complex process. Many factors, such as the digestive and circulatory systems, affect glucose uptake. Therefore, in vivo models are more reliable than cell lines. For this purpose, Zebrafish (*Danio rerio*) and its larvae have been developed as a convenient whole organism for drug screening [20,21]. Zebrafish share a similar mechanism to humans for glucose-homeostasis regulation. The larvae have a small enough size suitable for screening in 96-well microplates and have a transparent body under PTU exposure. Thus, we applied Zebrafish larvae to evaluate the glucose-uptake promotion of separated fractions. Moreover, fluorescent probe 2-NBDG was commonly used as a glucose indicator [22] in Zebrafish [7] or cell lines [23]. Thus, the Zebrafish larvae associated with 2-NBDG provide a convenient platform for activity-guided separation of functional natural products.

**Figure 1:**
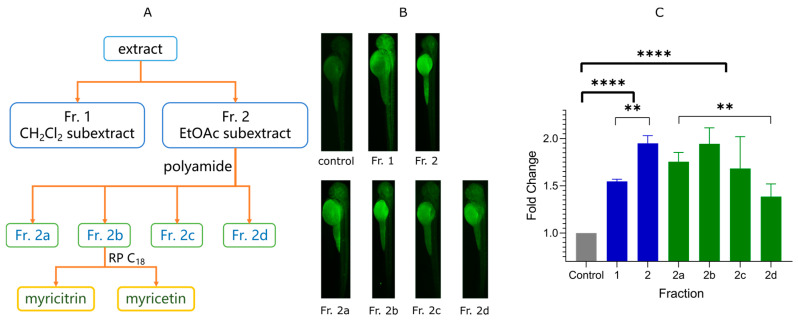
Bio-guided separation of the active components from *Acer truncatum* leaves. (**A**) Flow chart of bio-guided separation. (**B**) Fluorescent microscopy images after treatment of separated fractions. (**C**) Fold change of 2-NBDG fluorescence measured by ImageJ (** *p* < 0.01; **** *p* < 0.0001). The fold changes were calculated from the mean of the blank control. Green and blue are two different batches. The higher fold change indicates the better promotion of glucose uptake.

**Figure 2:**
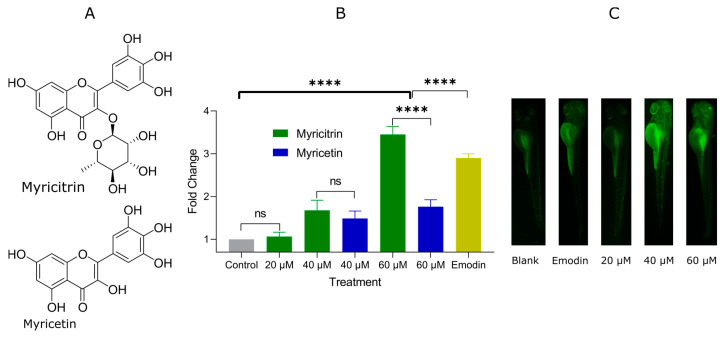
Myricitrin and myricetin-induced 2-NBDG uptake. (**A**) Chemical structures of myricitrin and myricetin. The structures were identified on the basis of HR MS/MS (Figure S2) and NMR spectra (Figure S3). (**B**) Fold change of 2-NBDG fluorescence measured by ImageJ (**** *p* < 0.001, ns, no statistical difference). The higher fold change indicates the better promotion of glucose uptake. (**C**) Fluorescent microscopy images after treatment of myricitrin and emodin.

The EtOH extract of leaves of *A. truncatum* was separated in a bio-guided process, as shown in Figure 1A. Glucose-uptakes were traced and quantified using fluorescence intensity of 2-NBDG, which a fluorescent probe of glucose [24]. The fluorescence intensity can reflect 2-NBDG concentration in vivo, reflecting the ability of glucose transportation or uptake [22]. As shown in Figure 1B,C, Fr. 2 (EtOAc subextract) showed more fluorescence intensity than Fr. 1 (CH_2_Cl_2_ subextract), which indicated that Fr. 2 has a higher promotion for glucose uptake. We applied polyamide chromatography for further separation since we detected many polyphenols on the thin layer chromatography (TLC) and LC-MS/MS (Section 2.3 for details). The eluate **2b** showed more activity than others. Thus, a further purification focusing on Fr. **2b** affords two compounds, which chemical structures (Figure 2A) were identified as myricitrin and myricetin according to ^1^H, ^13^C NMR and HR ESI-MS data [25,26]. This is the first time to identify the essential components of anti-diabetes from the plant *A. truncatum*. HPLC profile of EtOH extract (Figure S1) showed that the leaves contain 0.17% myricitrin and 0.027% myricetin. The leaves of *A. truncatum* can be applied as a new plant source for myricitrin.

### 2.2. Glucose Uptake Promotion of Myricitrin and Myricetin

To verify myricitrin and myricetin’s bioactivities, we then evaluated the dose-effect relationship of the two compounds for promoting glucose uptake. As shown in Figure 2B,C, myricitrin showed moderate promotion in a dose-dependent mode. Treating with 40 μM and 60 μM myricitrin enhanced the glucose uptake in larvae significantly. However, the aglycon myricetin showed lower effects than myricitrin, especially at 60 μM level (*p* < 0.0001). The rhamnose might be an essential pharmacophore for glucose-uptake regulation. Furthermore, 60 μM myricitrin treatment afford better promotion of glucose absorption than that of positive control emodin (*p* < 0.0001). Emodin is the main component in the traditional medicine *Rehmannia glutinosa*, which has a purgative effect and is not so suitable for long-term use. However, myricitrin and myricetin are also contained in the fruit waxberry (*Myrica rubra*), so they are safe enough even to develop health products. In a word, Myricitrin and myricetin are essential insulin mimics in *A. truncatum* leaves. This result also rationalized the hypoglycemic effect of myricitrin [27] from the glucose transportation aspect.

### 2.3. LC-MS/MS Profiling of Leaves Components form Different Age Trees

LC-MS/MS has been a crucial tool for analyzing small metabolites. It has a high sensitivity to detect trace substances but also can determine chemical structures simultaneously. Furthermore, with the continuous improvement of reference databases and data mining methods, LC-MS/MS provides more and more reliable analysis in metabolomics [28,29], which is often used in exploring biological metabolites [30] and natural products in traditional medicines or foods [31,32].

**Figure 3:**
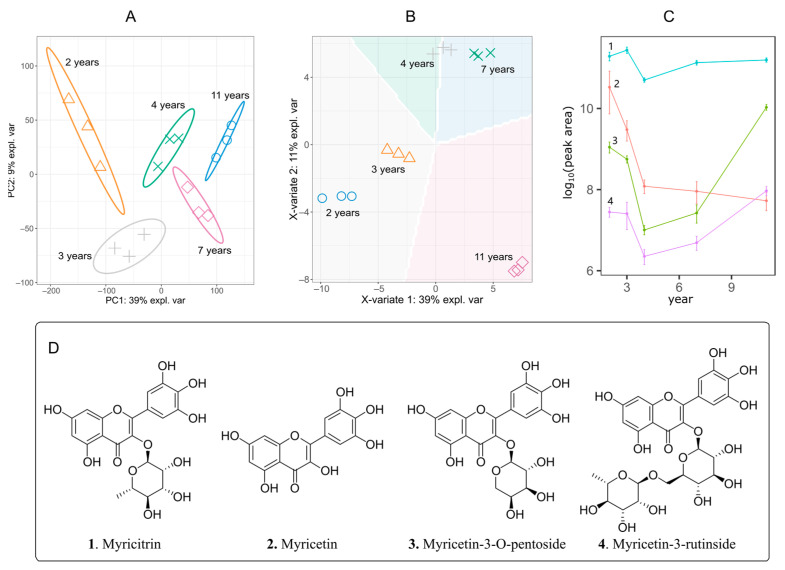
LC-MS/MS features of leaves from different years old trees (*A. truncatum*). (**A**) PCA analysis; (**B**) sPLS-DA analysis with multivariate prediction. (**C**) Content change trend of myricitrin-like components. 1-myricitrin; 2-myricetin; 3-myricetin-3-*O*-pentoside; 4-myricetin-3-rutinoside. Peak areas were calibrated with an external standard myricitrin. (**D**) Chemical structures of myricitrin-like components.

*A. truncatum* is a perennial tree that is widely planted in many places. However, it is still unclear whether tree age affects the accumulation of active ingredients in leaves. So, how many years after planting, can the active ingredients in the leaves reach the optimal level? We collected fresh leaves randomly from trees of different growth years (2, 3, 4, 7 and 11 years) from a plantation in Shaanxi. The LC-MS/MS scan yielded 6269 chemical features, among which 1790 features were identified based on the MS/MS matched. Reducing dimensionality is generally used to represent the differences of too many features in a 2D coordinate system, such as principal component analysis (PCA) [33] and Sparse PLS discriminant analysis (sPLS-DA) [34]. All the quantitative information of components and samples was gathered and analyzed by PCA (Figure 3A) and sPLS-DA (Figure 3B). PCA is a standard dimension reduction analysis method that can capture the differences among many features of different groups. The PCA analysis (Figure 3A) of all samples indicated five aged groups have significant variances from each other. sPLS-DA is a standard and powerful method of partial least squares regression to reduce the dimensions of data. At the same time, it establishes the regression model and carries on the discriminant analysis to the regression results. sPLS-DA principal component analysis can be combined with the experimental group to give variance prediction information. As shown in Figure 3B, the leaf components of 2-year-old and 3-year-old trees had a smaller difference, while 4-year-old and 7-year-old trees were close. After the trees grew up for 11 years, the leaf ingredients changed significantly. In short, tree age has a significant effect on leaf components. The larger the annual span was, the more pronounced the impact was (Figure 3A,B).

We then focused on those small molecules highly related to the active ingredient myricitrin. Thus, we used the RDkit package to deal with a large number of the identified structures by LC-MS/MS. Substructure searching found four molecules (myricitrin, myricetin, myricetin-3-*O*-pentoside, and myricetin-3-rutinoside) possessed the moiety of myricetin (Figure 3D). Their chemical relationship indicated that they might be generated from the same biosynthesis pathway. We extract their content information associated with tree ages. The relative contents were evaluated on the basis of peak areas which were calibrated by an external standard myricitrin. As shown in Figure 3C, the content of the active myricitrin was higher than the other three molecules and kept stable in different tree age samples. The contents of glycosides (myricetin-3-*O*-pentoside and myricetin-3-rutinoside) rose after 4 years, while the content of the aglycon (myricetin) decreased dramatically. Since myricitrin showed more activity than other components in *A. truncatum* leaves, these changing trends indicated that only myricitrin could serve as a marker of quality evaluation for the hyperglycemic effect *A. truncatum* leaves.

**Figure 4:**
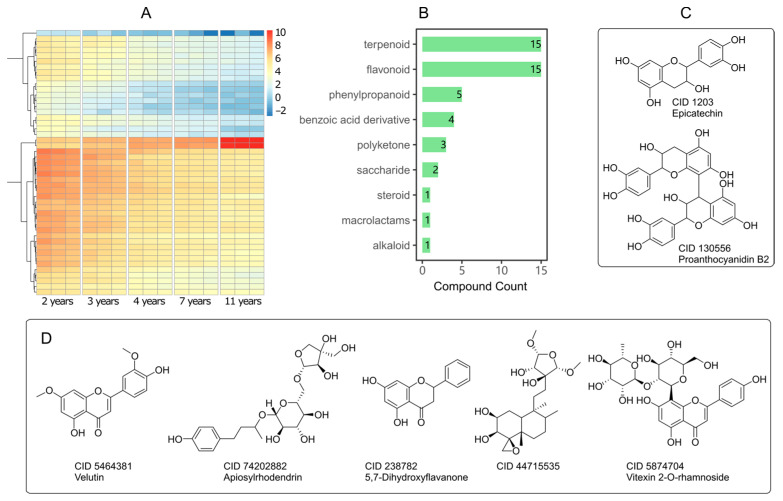
Main components in leaves affected by tree ages. (**A**) Hot spot map of increasing and decreasing components depending on tree age. The color palette represents the content of metabolites. (**B**) Components counts in different structural types. (**C**) Compounds up-regulated annually (FC > 32, *p* < 0.01). (**D**) Representative compounds down-regulated annually (FC > 32, *p* < 0.01). See Table S1 for details.

To filter out key components related to tree ages, we select the components whose mean value increases or decreases following tree ages strictly. The change fold (FC) was calculated with maximum/minimum mean values. Those components with FC > 2 and statistical *p*-value < 0.01 were selected as the significant change components. Among them, 47 components can be annotated by MS/MS matching (score > 80). Their changing trend was shown in Figure 4A. All these changing 47 components belonged to 9 structural types, mainly flavonoids and terpenoids (Figure 4B). Only two ingredients (Figure 4C) increased with tree ages dramatically (FC > 32). The other 45 ingredients decreased following tree growing. Five of them (Figure 4D) changed 32-fold from 2-year to 11-year-old trees. The chemical details and the change information of these components following tree ages were listed in Table S1 in the Supplementary Materials. In brief, tree age affected the diversity and content of leaf components, especially terpenoids and flavonoids. However, the key active component myricitrin was not affected by this. In terms of hypoglycemic function, leaves form it in 2-year-old trees, and 11-year-old trees have the same effect.

### 2.4. Pathway of Myricitrn Effects Based on Transcriptomic and Metabolomic Investigation

It was evident that myricitrin is the essential component for the hypoglycemic function of *A. truncatum* leaves. Since the leaves of *A. truncatum* are considered potential natural sources for preventing diabetes development, it is necessary to clarify the primary mechanism for the hypoglycemic effect. Regulations of natural products often have multi-targets. We prefer to use omics methods, such as transcriptome and metabolome analysis, to clarify the main regulatory network related to myricitrin regulation and diabetes disorder. Although myricitrin was reported to cause hyperglycemic effects in diabetes mice [27], the main regulatory pathways, especially the transcriptome and metabolome responses, were still waiting for exploration. Herein, we designed three groups: blank control, model group and test (myricitrin administration) group. The blank control was maintained with only normal E3 water. The model and test groups were treated with alloxan and sucrose to destroy β-cells to form glucose transport abnormity. The test group was given myricitrin in addition.

**Figure 5:**
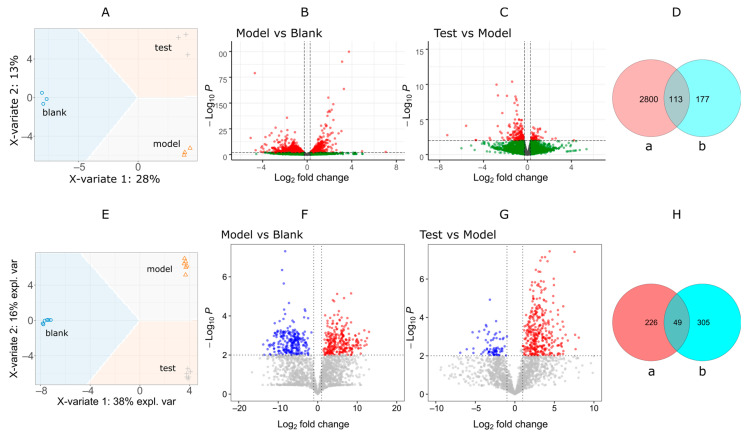
Visual analysis of differentially expressed genes (DEGs) and differential metabolites (DMs). (**A**) sPLS-DA analysis of DEGs. (**B**,**C**) valcono plot of DEGs (*p* < 0.01). (**D**) Venn plots of DEG counts. a, model vs. blank; b, test vs. model. (**E**) sPLS-DA analysis of DMs. (**F**,**G**) valcono plot of DMs (FC > 2, *p* < 0.01). (**H**) Venn plots of DM counts. a, model vs. blank; b, test vs. model.

After RNA-seq, quality control and gene annotation, 24,780 mRNA in total were obtained. We applied sPLS-DA for the samples’ variance inspection since PLS is a supervised algorithm that can better select the characteristic variables to distinguish each group and determine the relationship among samples. The three groups clustered along with the biological treatments from the sPLD-DA analysis, as shown in Figure 5A for RNA-seq samples. The differentially expressed genes (DEGs) pairwise from the three groups were filtered out with a statistical *p*-value < 0.01, as shown in the volcano plots in Figure 5B,C. The counts of DEGs were gathered in the Venn diagram (Figure 5D). There were 113 DEGs (see Table S2 for detailed list) in the intersection of the “model vs. blank” set and “test vs. model” set. They were highly related to the diabetic development invoked by alloxan and myricitrin curation.

Metabolites are effective marks reflecting biological phenotype and state. It can track various dynamic responses in living organisms to external stimulations. Here, we used LC-MS/MS to monitor the metabolites changes among the three groups to search for more evidence related to myricitrin regulations. The sPLS-DA plot (Figure 5E) shows a total difference of 54% in both vertical and horizontal directions. The different treatments to Zebrafish afford noticeable changes of the metabolome. Thus, it is feasible to find the differential metabolites (DM) related to the biological procedure. The volcano (Figure 5F,G) and Venn plots (Figure 5H) showed that 49 differential metabolites (DMs) were filtered out with standard criteria (FC > 2 and *p* < 0.01).

**Figure 6:**
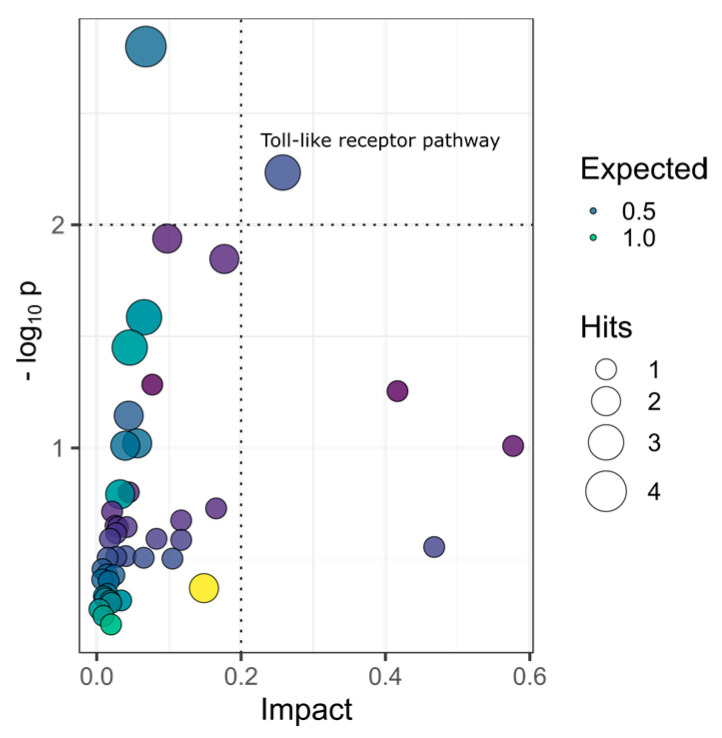
KEGG pathway analysis based on combining DEGs and DMs.

The DEGs and DMs were submitted to the MetaboAnalyst online to perform enrichment and joint pathway analysis associated with the KEGG database. According to the path impact and p-value, the toll-like receptor pathway is the greatest affected pathway, as shown in Figure 6 and Table 1. In the DEGs and DMs filtering, we focus on genes and metabolites related to both diabetes development and myricitrin effects. In combination with the phenotypic activity results, myricitrin can promote glucose absorption and reduce blood glucose levels by affecting the toll-like receptor pathway. Although myricitrin can influence MAPK and PPAR pathways in the enrichment, however, the impacts and *p*-values are very limited.

### 2.5. qRT-PCR Verification

**Figure 7:**
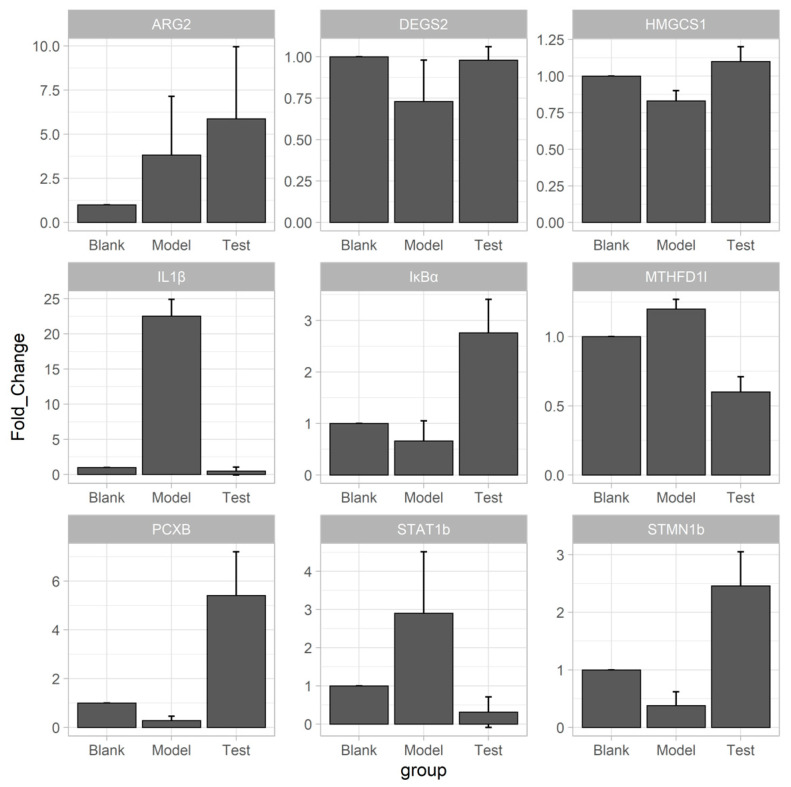
RT-qPCR validation of DEGs related to myricitrin’s regulation.

All the feature genes (Table 1) were verified by qPCR furtherly. The fold changes of expression in qPCR indicated that some feature genes changed reversely in the model and test groups (Figure 7). The genes (PCXB, HMGCS1, DEGS2, IκBα and STMN1b) down-regulated in the model group and then up-regulated in the test group. Other genes, such as STAT1b, IL1b and MTHFD1l, up-regulated in the model group and down-regulated in the test group. The most obviously changed gene was IL1β among the three groups. After treated with alloxan, the expression in the model group increased by 24.8 times compared to the blank control and then decreased to 1.1-fold after myricitrin administration. These reverse FCs indicated that the treatment with myricitrin curated the destruction of gene expression by alloxan or diabetes development. IκBα, STMN1b and IL1b were featured with toll-like receptor pathways among these changed genes. The toll-like receptor can sensor β-cell death [35]. Moreover, blocking the toll-like receptors can slow down β-cell death in diabetes [36]. This pathway also plays an essential role in diabetes renal injuries [37] and insulin resistance [38].

## 3. Materials and Methods

### 3.1. General

NMR spectra were recorded in MeOD using an AVANCE III spectrometer (400 MHz). The chemical shifts (in ppm) referred to the solvent residue. Coupling constants (*J*) were recorded in the Hz unit. LC-MS/MS data were read on an AB Sciex Triple TOF 5600 + instrument (AB Sciex Pte. Ltd., Framingham, MA, USA). 2-NBDG was purchased from Dibai Biotechnology Co. Ltd. Shanghai, China. Fluorescent photos were captured on a Nikon fluorescence stereomicroscope SMZ25 (Nikon Corp, Tokyo, Japan).

### 3.2. Bio-Guided Isolation of Myricitrin

The leaves of trees of different ages were mixed together (3 Kg), which was ground and extracted by EtOH to yield 257.3 g crude extract after the solvent was evaporated on a rotavapor under vacuum and 45 °C temperature. Then the crude extract was dispersed into the water, which was extracted, respectively, by CH_2_Cl_2_ and EtOAc three times to afford Fractions **1** (20.6 g) and **2** (62.8 g). Fraction **2** was submitted to a polyamide column chromatography, which eluted with 30%, 50%, 70% and 100% MeOH to afford fractions **1a**–**1d**. Further purification of 500 mg Fraction **2b** on reverse phase C_18_ column with 35% MeOH to afford two pure compounds, myricitrin and (103 mg) and myricetin (32mg). Their chemical structures were identified by comprising ^1^H, ^13^C NMR, and high-resolution(HR) ESI-MS [25,26].

#### 3.2.1. Myricitrin

^1^H NMR (400 MHz, MeOD) *δ* ppm 0.93 (d, *J* = 6.0 Hz, 3 H) 3.28–3.36 (m, 1 H), 3.44–3.56 (m, 1 H), 3.76 (dd, *J* = 9.4, 2.9 Hz, 1 H), 4.19 (br. s., 1 H), 5.28 (s, 1 H), 6.16 (s, 1 H), 6.32 (s, 4 H) and 6.91 (s, 2 H); ^13^C NMR (100 MHz, MeOD) *δ* ppm 17.8, 72.0, 72.2, 72.2, 73.5, 94.8, 99.9, 103.7, 106.0, 109.7, 122.0, 136.4, 138.0, 147.0, 158.6, 159.6, 163.3, 166.0 and 179.8. HR ESI-MS *m*/*z* Full MS [M + H]^+^ calcd for C_21_H_21_O_12_ 465.10330, found 465.09906; MS2, *m*/*z* 319.0443.

#### 3.2.2. Myricetin

^1^H NMR (400 MHz, MeOD) *δ* ppm 6.17 (d, *J* = 2.0 Hz, 1 H) 6.36 (d, *J* = 2.0 Hz, 1 H) 7.34 (s, 2 H); ^13^C NMR (100 MHz, MeOD) *δ* ppm 177.4, 165.7, 162.6, 158.3, 148.1, 146.8, 137.5, 137.0, 123.2, 108.6, 104.6, 99.3 and 94.5. HR ESI-MS *m*/*z* Full MS [M + H]^+^ calcd for C_15_H_11_O_8_ 319.04539, found 319.04385; MS2, *m*/*z* 273.0402, 153.0175.

### 3.3. HPLC-DAD Profiling of A. truncatum Leaves

The above extract solution in methanol (1.0 mg/mL) was filtered with a 0.22 μm filter and then analyzed on an Agilent 1100 HPLC (Agilent Technologies, Inc., Santa Clara, CA, USA). The sample was separated on a column of XBridge Shield RP 18 (5 μm, 4.6 mm × 250 mm), eluted with 10–100% methanol in 30 min. The flow rate was 1.0 mL/min, and the injection volume was 5 μL. DAD inspector was set at 190–400 nm. Myricetin and myricitrin (1 mg/mL) were used as external standards.

### 3.4. Zebrafish Maintenance

The Zebrafish and the eggs (wild AB type) were maintained according to the reported method [8]. In brief, the female and male adults were randomly selected at a ratio of 1:1 and separated by a board in a spawning tank. On the next day, the Zebrafish eggs were collected and incubated in E3 water (5 mM NaCl, 330 µM CaCl_2_ and 330 µM MgSO_4_, 170 µM KCl) at 28 °C. The 2-Phenylthiourea (PTU, 0.2 mM) was then added to the culture water to increase body transparency. Culture water was changed daily.

### 3.5. Insulin Mimetic Bioassay on Zebrafish Larvae

The 2-NBDG uptake fluorescence was captured as a reported description [7,8] with slight modification. Briefly, each well of 96-well plate was placed into five 72 hpf (hour of post-fertilization) larvae with 200 μL E3 water containing test compounds. The larvae were maintained for 1 h and then treated with 2-NBDG (0.6 mM) for a further 3 h. After being washed using E3 water and anesthetized using 0.25% tricaine, the larvae were captured on fluorescence microscopy, and the uptake of 2-NBDG was quantified using Fiji (ImageJ) following a standard method [39].

### 3.6. LC-MS/MS Analysis of A. truncatum Leaves

We collected *A. truncatum* leaves from trees of five different ages in Jinshan Agricultural Technology Co., Ltd. (East longitude 108.09 and north latitude 34.32, Yangling, China). Three leaf samples were collected from three randomly selected trees in each age (2, 3, 4, 7 and 11 years old). The fresh leaves (10 g each sample) were ground and dipped in methanol on site. After overnight extraction, the samples were treated with ultrasonic at 40 °C for 2 h. The solvents were removed on a rotary evaporator, and the residues were made into 100 μg/mL solutions. Myricitrin (1.0 μg/mL and 10.0 μg/mL) was used as external standard. Then the solution samples were analyzed on an AB SCIX 5600 + instrument. Each sample (2 μL) was loaded and separated on a UPLC C_18_ column (Shim-pack XR-ODS, 2.0 mm × 100 mm). The column was eluted by gradient MeOH 10–100% associated with 0.1% HCOOH in 18 min. The full MS was scanned from 100 *m*/*z* to 1000 *m*/*z*. The collision energy was set to 35 V, and fragments were scanned by the SWATH method. The raw data files (wiff) were transferred by Reifycs Abf Converter and then imported into the open-source tool MS-Dial 4.48 [40]. After alignment, identification, and normalization in MS-Dial, the data matrix (including MS features, metabolite identifications, normalized peak areas) was exported for further analysis. PCA and sPLS-DA were analyzed and visualized by mixOmics following official instructions (http://www.bioconductor.org/packages/release/bioc/vignettes/mixOmics/inst/doc/vignette.html, accessed on 9 May 2021). A hot spot map was drawn using the R package pheatmap. Similar components to myricitrin were searched out based on SMART strings by an open-source package RDKit.

### 3.7. Zebrafish Larvae RNA Sequencing and Data Analysis

The 72 hpf Zebrafish larvae (540 in total) were divided into three groups with three biological replicates (60 larvae in each replicate). The three groups were set as blank control, diabetes model, and test (myricitrin administration) groups. The blank group was only maintained in daily fresh E3 water. The model group was treated with 4% sucrose and 0.2 M alloxan to induce diabetes status, and the test group had 60 μM more myricitrin than the model group.

The treated larvae were collected and packaged with dry ice, then shipped to Beijing Genomics Institution (Wuhan, China) for RNA-seq. Total RNA was extracted from the larvae with a Trizol kit, then qualified and quantified on a NanoDrop and Agilent 2100 bioanalyzer (Thermo Fisher Scientific, Waltham, MA, USA). After being purified with Oligo(dT) beads, mRNA was fragmented for reverse transcription to cDNA. Single-end 50 bases reads were generated on the BGISEQ500 platform. Sequencing data was filtered using SOAPnuke (v1.5.2) [41] to remove adapters and lower-quality bases. The clean reads were mapped by HISAT2 (v2.0.4) [42] and aligned by Bowtie2 (v2.2.5) [43] to reference the genome. Gene expression were evaluated by RSEM (v1.2.12) [44]. Differentially expressed genes (DEGs) were filtered out using DESeq2 (v1.30.1) [45] with the threshold of *p*-value < 0.01.

### 3.8. Zebrafish Larvae Metabonomics Analysis

Samples for metabonomic analysis were prepared following the protocol for the transcriptomic samples in parallel. We collected 100 larvae for each replicate to ensure enough extract for LC-MS/MS analysis. After being washed with freshwater, the metabolites in each replicate were extracted by 350 μL MeOH (2:1, *v*/*v*) at 4 °C. The metabolites were analyzed on a UPLC-qTOF-ESIMS system (AB SCIEX 5600+). Each sample was injected 5 μL. The metabolites were separated on a HILIC column (ACQUITY BEH Amide 1.7 μm, 2.1 mm × 100 mm), eluted by a gradient MeCN (90–40% with 0.1% AcOH) with the flow rate of 0.3 mL/min. The full MS was scanned from 50 *m*/*z* to 1000 *m*/*z*. The MS/MS data was acquired by a DIA SWATH method.

LC-MS/MS raw data files (*.wiff) were transformed into ABF files by Reifycs ABF converter and imported into MS-Dial for further analysis. The parameters were set according to the experimental design, including error range, retention time range and adduct type. The data of each replicate was aligned and normalized according to the TIC. Then the normalized matrix (including MS features, metabolite identifications, and normalized peak areas) was export as a CSV file for further analysis. The sPLS-DA was analyzed and visualized using the R package mixOmics. Fold change was calculated on the basis of normalized peak area. P-values were calculated using the oneway test function in R. The volcano plots were drawn by R package ggplot2, and the Venn plots were drawn by R package ggvenn. The differential metabolites (blank vs. model and model vs. test) were filtered with the threshold of Fold Change (FC) > 2 and *p*-value < 0.01. Then the shared differential metabolites were selected as the critical metabolite to perform KEGG enrich and pathway analysis. The enrichment result was visualized in a bubble plot using ggplot2.

### 3.9. qRT-PCR Analysis

To further verify myricetin’s effect on the gene expression involving diabetes metabolic recovery, we gathered 100 Zebrafish larvae from each group of blank control, model and test after two days of culture. According to the product instructions, total RNA was extracted using AG RNAex Pro Reagent (Accurate biology, Changsha, China). The extracted RNA was reverse transcribed with Evo M-MLV (RT kit with gDNA clean for qPCR II). RT-PCR was performed on a CFX 96 touch instrument (Bio-Rad Laboratories, Inc., Hercules, CA, USA). Primer sequences and the reference sequence (β-actin) are listed in Supplementary Materials (Table S3). Three biological replicates with three PCR replicates were carried out for each sample. The qPCR protocol included annealing at 95 °C for 30 s, followed by 40 cycles at 95 °C for 5 s and 60 °C for 30 s. Relative expression levels were evaluated based on the 2^−ΔΔCt^ and normalized to the reference gene [46].

## 4. Conclusions

In conclusion, we isolated and identified the active substances from *A. truncatum* leaves guided by a whole organism bioassay. They showed obvious effects on promoting glucose uptake. The glycoside (myricitrin) was more active than the aglycone (myricetin). Food resource origin indicated they were high safe for long-term use in preventing early diabetes development. The essential active compound myricitrin showed stable contents in leaves of trees of different ages (2–11 years old). Therefore, myricitrin can also be used as a marker ingredient to evaluate product qualities derived from *A. truncatum* leaves. Although many flavonoids and terpenoids in the leaves decreased with trees growing, they are unrelated to the anti-diabetes function. Omics investigations disclose the whole transcriptome and metabolome response to myricitrin. Myricitrin can repair abnormal expression of mRNA IL1β, STAT1b and IκBα in diabetes disorder, thus mainly interfered with toll-like receptor pathway to promote glucose uptake and relieve hyperglycemia level in vivo. Myricitrin was reported to ameliorate hyperglycemia and glucose intolerance in diabetic mice [27]. This also confirmed that our findings of glucose-uptake promotion could evoke an anti-diabetic effect. The leaves of *A. truncatum* or myricitrin could be used for developing healthy products to prevent diabetes development. However, pharmacokinetic characteristics are waiting for further exploration.

## Figures and Tables

**Table 1:** Matched features in KEGG pathways analysis.

Pathway	−logP	Impact	Matched Features
One carbon pool by folate	1.01	0.58	MTHFD1l
Sphingolipid metabolism	0.57	0.47	DEGS2
Synthesis and degradation of ketone bodies	1.25	0.42	HMGCS1
Toll-like receptor signaling pathway	2.23	0.26	IL1β; STAT1b; IκBα
Arginine biosynthesis	1.85	0.18	ARG2, L-Ornithine
Citrate cycle (TCA cycle)	0.73	0.16	PCXB
MAPK signaling pathway	0.37	0.15	STMN1b; IL1β
PPAR signaling pathway	0.59	0.12	HMGCS1
RIG-I-like receptor signaling pathway	0.68	0.12	IκBα
Adipocytokine signaling pathway	0.50	0.10	IκBα

## Data Availability

Data is contained within the article and supplementary material.

## Supplementary Materials

The following are available online at https://www.mdpi.com/article/10.3390/ph14070662/s1, Table S1: Leaves components changing with tree ages, Table S2: Differentially Expressed Genes (DEGs), Table S3: Primers used for qPCR analysis. Figure S1: HPLC profile of *A. truncatum* leaves (EtOH extract), Figure S2: HR ESI-MS/MS of myricitrin-like components, Figure S3: ^1^H (400 MHz) and ^13^C (100 MHz) NMR spectra.
