# Supplementary material for: Insulin-Mimic Components in Acer truncatum Leaves: Bio-Guided Isolation, Annual Variance Profiling and Regulating Pathway Investigated by Omics
Source: Pharmaceuticals (Basel). 2021 Jul 11;14(7):662. doi: 10.3390/ph14070662 (PMC8308865; doi:10.3390/ph14070662)
Supplement: Supplementary file 1 [file pharmaceuticals-14-00662-s001.zip › pharmaceuticals-1287165-supplementary.pdf]

SUPPLEMENTARY MATERIAL

Insulin-mimic Components from *Acer truncatum* leaves: Bio-guided Isolation, Annual-Variance Profiling and Regulating Pathway Investigated by Omics

Xiao-Yue Zhang [1], Yi-Han Liu [1], Da-Zhi Liu [1], Jia-Yang Xu [1] and Qiang Zhang [1,*]

Shaanxi Key Laboratory of Natural Products & Chemical Biology, College of Chemistry & Pharmacy, Northwest A&F University, Yangling, China, e-mail: zhangq@nwsuaf.edu.cn

Contents

Abbreviation List 2

Table S1. Leaves components changing with tree ages 3

Table S2. Differentially Expressed Genes (DEGs) 5

Table S3 Primers used for qPCR analysis. 6

Figure S1. HPLC profile of *A. truncatum* leaves (EtOH extract) 6

Figure S2. HR ESI-MS/MS of myricitrin-like components 7

- A. Myricitrin (CID 5281673) 7
- B. Myricetin (CID 5281672) 7
- C. Myricetin-3-O-pentoside (CID 21477996) 8
- D. Myricetin-3-rutinside (CID 73803273) 8

Figure S3. ^{1}H (400 MHz) and ^{13}C (100 MHz) NMR spectra 9

- A. Myricitrin 9
- B. Myricetin 10

Abbreviation List

DEGs, differentially expressed genes

DMs, differentially metabolites

FC, Fold Change

KEGG, Kyoto Encyclopedia of Genes and Genomes

LC-MS/MS, Liquid Chromatography coupled to tandem Mass Spectrometry

2-NBDG, 2-deoxy-2-[(7-nitro-2,1,3-benzoxadiazol-4-yl) amino]-D-glucose

PTU, 2-Phenylthiourea

PCA, Principal Component Analysis

qRT-PCR, Reverse Transcription−Quantitative Real-Time Polymerase Chain Reaction

sPLS-DA, Sparse Partial Least Squares-Discriminant Analysis

T2DM, Type 2 diabetes mellitus

TLC, Thin Layer Chromatography

TIC, Total Ions Chromatography

Table S1. Leaves components changing with tree ages

	CID[a]	Rt (min)	Score[b]	MF	Class	log2 FC	– lg p
**1**	5464381	6.600	83.9	$C_{17}H_{14}O_6$	flavanonoid	– 5.57	3.68
**2**	74202882	10.220	82.6	$C_{21}H_{32}O_{11}$	phenylpropanoid	– 5.20	4.17
**3**	238782	11.244	80.5	$C_{15}H_{12}O_4$	flavanonoid	– 5.18	3.09
**4**	44715535	6.611	80.0	$C_{22}H_{38}O_8$	terpenoid	– 4.99	3.50
**5**	5874704	8.579	88.5	$C_{27}H_{30}O_{14}$	flavanonoid	– 4.90	3.23
**6**	4635494	5.026	82.4	$C_{17}H_{20}O_9$	phenylpropanoid	– 4.83	3.78
**7**	445858	5.033	85.3	$C_{10}H_{10}O_4$	phenylpropanoid	– 4.68	4.41
**8**	872	0.741	81.6	$C_{12}H_{22}O_{11}$	saccharide	– 4.59	4.47
**9**	188366	10.962	86.8	$C_{18}H_{28}O_3$	polyketone	– 4.55	3.32
**10**	3789874	5.504	83.9	$C_{25}H_{32}O_{13}$	terpenoid	– 4.51	3.75
**11**	75111043	12.374	87.8	$C_{22}H_{32}O_6$	terpenoid	– 4.48	3.44
**12**	78124326	13.928	93.2	$C_{29}H_{30}O_{11}$	phenylpropanoid	– 4.31	4.67
**13**	85503083	8.871	90.7	$C_{22}H_{22}O_{11}$	flavanonoid	– 4.30	4.55
**14**	13245586	8.134	94.3	$C_{22}H_{22}O_{12}$	flavanonoid	– 4.27	3.16
**15**	3705071	15.459	84.6	$C_{48}H_{59}N_3O_{13}$	macrolactams	– 4.26	3.98
**16**	74977425	13.975	93.0	$C_{27}H_{30}O_{14}$	flavanonoid	– 4.26	5.55
**17**	273110	5.538	89.9	$C_{17}H_{24}O_{10}$	terpenoid	– 4.25	3.63
**18**	431673	14.126	80.2	$C_{20}H_{27}NO_3$	terpenoid	– 4.23	4.41
**19**	5281672	6.668	83.8	$C_{15}H_{10}O_8$	flavanonoid	– 4.22	3.01
**20**	435242	11.244	88.3	$C_{22}H_{33}NO_4$	terpenoid	– 4.20	4.34
**21**	11092	12.092	80.9	$C_9H_{10}O_3$	benzoic acid derivative	– 4.18	3.74
**22**	5280459	7.273	92.6	$C_{21}H_{20}O_{11}$	flavanonoid	– 4.17	3.03
**23**	15625347	10.956	84.6	$C_{46}H_{74}O_{16}$	terpenoid	– 4.17	4.58
**24**	13892721	10.491	84.7	$C_{17}H_{26}O_{11}$	terpenoid	– 4.15	3.53
**25**	5280804	5.482	85.8	$C_{21}H_{20}O_{12}$	flavanonoid	– 4.15	3.14
**26**	5281654	8.871	90.6	$C_{16}H_{12}O_7$	flavanonoid	– 4.11	4.01
**27**	5099	3.600	86.4	$C_{18}H_{16}O_8$	phenylpropanoid	– 4.00	3.50
**28**	76401267	7.346	88.5	$C_{16}H_{28}O_7$	terpenoid	– 4.00	3.20
**29**	607	12.600	81.6	$C_6H_{10}O_8$	saccharide	– 3.94	4.49
**30**	12312977	11.696	81.4	$C_{17}H_{26}O_{11}$	terpenoid	– 3.93	3.99
**31**	5280961	15.030	86.3	$C_{15}H_{10}O_5$	flavanonoid	– 3.90	4.00
**32**	633458	14.366	89.1	$C_{24}H_{34}O_5$	steroid	– 3.85	5.28
**33**	72	10.417	85.6	$C_7H_6O_4$	benzoic acid derivative	– 3.84	4.71
**34**	170157	17.120	81.5	$C_{22}H_{35}NO_4$	terpenoid	– 3.69	3.28
**35**	5280805	5.482	91.2	$C_{27}H_{30}O_{16}$	flavanonoid	– 3.67	3.64
**36**	4961358	15.143	92.4	$C_{14}H_{20}O_7$	benzoic acid derivative	– 3.52	4.47
**37**	321937	17.229	80.0	$C_{37}H_{40}N_2O_6$	alkaloid	– 3.42	4.44
**38**	69604805	5.371	92.9	$C_{12}H_{18}O_4$	polyketone	– 3.32	3.09
**39**	8214	17.007	81.9	$C_{22}H_{43}NO$	polyketone	– 3.13	4.81
**40**	3084407	5.651	91.4	$C_{27}H_{30}O_{15}$	flavanonoid	– 3.06	3.25
**41**	14334	6.668	92.0	$C_{11}H_{16}O_3$	terpenoid	– 3.01	3.43
**42**	133052557	16.609	86.7	$C_{24}H_{38}O_4$	benzoic acid derivative	– 2.86	5.23

**43**	3133561	17.233	87.9	$C_{22}H_{33}NO_3$	terpenoid	– 2.61	4.80
**44**	3917786	12.035	81.3	$C_{15}H_{22}O_{10}$	terpenoid	– 2.57	6.64
**45**	76401242	7.318	80.0	$C_{21}H_{28}O_9$	terpenoid	– 2.34	3.30
**46**	1203	4.352	93.9	$C_{15}H_{14}O_6$	flavanonoid	5.54	11.27
**47**	130556	4.182	84.6	$C_{30}H_{26}O_{12}$	flavanonoid	6.01	13.48

[a] CID, pubchem cid. All the structures can be acquired directly from PubChem (https://pubchem.ncbi.nlm.nih.gov) by CID.

[b] Score, identification score based on MS1 and MS/MS comparision in MS-Dial.

Table S2. Differentially Expressed Genes (DEGs)

#	NCBI ID	Name	#	NCBI ID	Name	#	NCBI ID	Name
1	797361	gngt2b	41	393758	pde6ha	81	561376	pus7l
2	563341	klhl38b	42	110438817	LOC110438817	82	405770	il1b
3	58068	pcxb	43	103910098	LOC103910098	83	566685	arrdc3a
4	100037332	zgc:162608	44	562666	cluha	84	558470	crygm2d5
5	794872	pcolceb	45	415223	trim63a	85	100149084	LOC100149084
6	100151239	fam89b	46	415228	crygm2d6	86	403009	ezra
7	100002200	barx2	47	550548	stmn1b	87	393891	fbxo32
8	568656	LOC568656	48	553474	crybgx	88	553584	endou2
9	322614	arg2	49	445319	ncaldb	89	550441	asb5a
10	406307	rps28	50	100329462	mfn1a	90	407681	foxf2a
11	386643	pik3ip1	51	550429	steap4	91	403309	rtn4rl2b
12	386968	calcoco1b	52	360151	opn1mw2	92	368481	stat1b
13	445040	rpl29	53	792160	irf1b	93	100007488	LOC100007488
14	558389	crygm2f	54	402799	degs2	94	393818	rps17
15	797707	crygm2d14	55	100330105	map1lc3cl	95	360145	mxc
16	393665	ndrg1b	56	561067	mxf	96	101884357	LOC101884357
17	493635	hapln1a	57	394060	hmgcs1	97	436855	crygm2d15
18	393725	rps7	58	100148329	LOC100148329	98	192300	rps18
19	406436	slc25a33	59	110437892	LOC110437892	99	436857	cryba2b
20	100334622	ubap1lb	60	436681	crygm2d13	100	405781	nfe2l1a
21	406760	eif4a2	61	558501	crygm2d2	101	541348	bccip
22	110438046	LOC110438046	62	393776	cox4i2	102	431765	nfil3-6
23	100331492	LOC100331492	63	553242	sh3pxd2b	103	555454	myh10
24	566659	si:ch211-176g13.7	64	555427	si:ch211-242e8.1	104	494573	guca1d
25	84703	slc4a1a	65	724003	rsrp1	105	564090	zgc:193593
26	101884530	dact3a	66	799956	si:dkeyp-72h1.1	106	402837	hamp
27	335409	socs3a	67	326961	rpl5a	107	794081	ucp3
28	393939	atf3	68	100006043	klf5a	108	559662	ppp1r27b
29	100000256	crygm2d12	69	100333206	LOC100333206	109	563472	si:rp71-39b20.4
30	103909923	103909923	70	570567	si:dkey-97a13.12	110	562950	si:dkey-7c18.24
31	561929	crygm2d17	71	100005729	urgcp	111	326864	ola1
32	30604	cfb	72	192299	rpl35	112	556563	si:dkey-7j14.5
33	100149559	cbln11	73	557397	rpl22	113	795575	si:dkey-57a22.15
34	792062	cyt1l	74	474321	pgm3			
35	101883708	LOC101883708	75	406463	nfkbiaa			
36	393520	pno1	76	568826	si:ch211-266i6.3			
37	799807	crygm2d9	77	100034522	mthfd1l			
38	794635	c4b	78	795305	acod1			
39	562682	si:dkey-251i10.2	79	795400	lss			
40	793897	psme4a	80	567192	cd59			

Table S3 Primers used for qPCR analysis.

#	NCBI ID	Gene name	Primer Name	Primer sequence
**1**	405770	il1b	il1b-F	5′- GATTCGCAGATGGTGGAGATGGAC -3′
			il1b-R	5′- TCGTCTTTGGATGGAAGCACAGC -3′
**2**	368481	stat1b	stat1b-F	5′- TCCTCCTCACAGTCCTAAGCACAG -3′
			stat1b-R	5′- ATCTGGGCGACGCTTCTATGTTTC -3′
**3**	322614	arg2	arg2-F	5′- CAGTTTCAGTTCCCGTCAGCAGAG -3′
			arg2-R	5′- CACTCCGCACCACATCACTTACAG -3′
**4**	402799	degs2	degs2-F	5′- TCTGGGTGGAGATGGCTTGGATG -3′
			degs2-R	5′- CTGGTCACGGGCTTTGGGTTG -3′
**5**	394060	hmgcs1	hmgcs1-F	5′- CACCACAGCAGTTAGCAGGACAG -3′
			hmgcs1-R	5′- CCAGGTGTGCCATCTTGAGTGAC -3′
**6**	100034522	mthfd1l	mthfd1l-F	5′- GTCTTCTCTGGCTGCTTCTCGTTG -3′
			mthfd1l-R	5′- ACGGTTTCCTCCCACATTCCAAAG -3′
**7**	58068	pcxb	pcxb-F	5′- AAGCTGGGATCTCTGGGCAT -3′
			pcxb-R	5′- CACTGCTGTCAACCTTGGCT -3′
**8**	406463	nfkbiaa	nfkbiaa-F	5′- CCTGACCTTCGGCAGACACAAC -3′
			nfkbiaa-R	5′- CGATTCGCTCTCTGGCATGACTG -3′
**9**	550548	stmn1b	stmn1b-F	5′- CAGAGCGGTCAGCCTGTGTTG -3′
			stmn1b-R	5′- TGCCAGCAGAAAACCCGAATCC -3′
**ref**		β-actin	β-actin-F	5′- CGTGCTGTCTTCCCATCCA -3′
			β-actin-R	5′- TCACCAACGTAGCTGTCTTTCTG -3′

Figure S1. HPLC profile of *A. truncatum* leaves (EtOH extract)

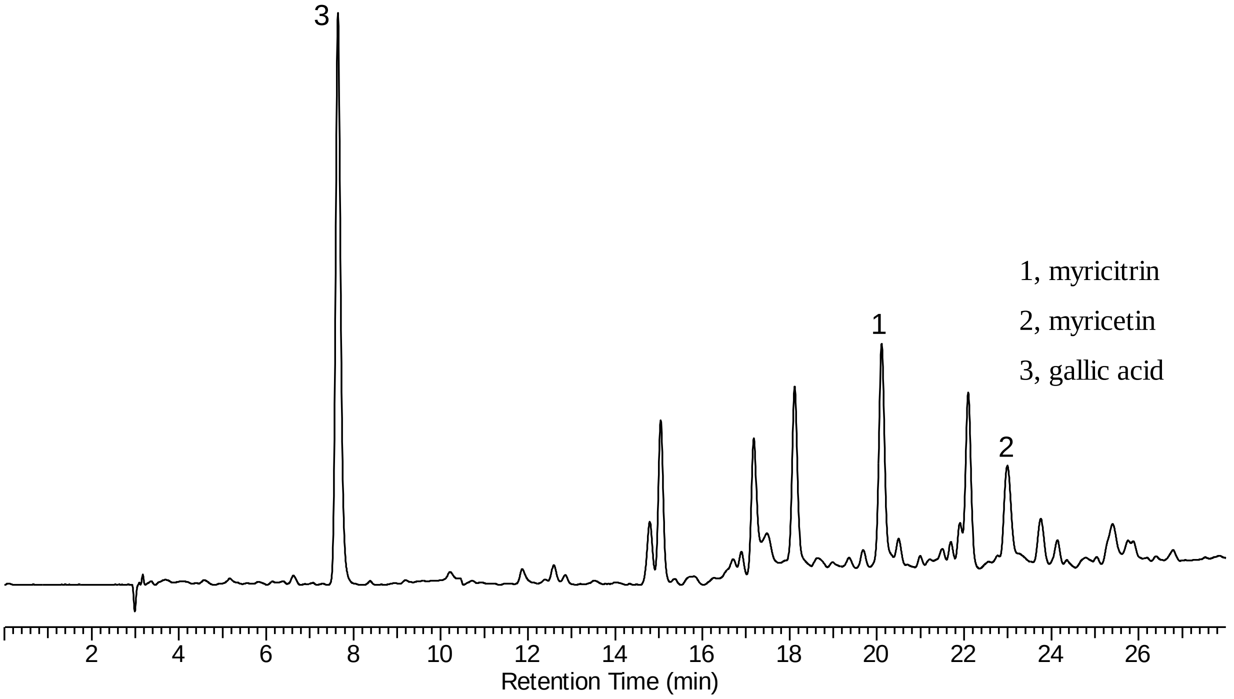

Figure S2. HR ESI-MS/MS of myricitrin-like components

A. Myricitrin (CID 5281673)

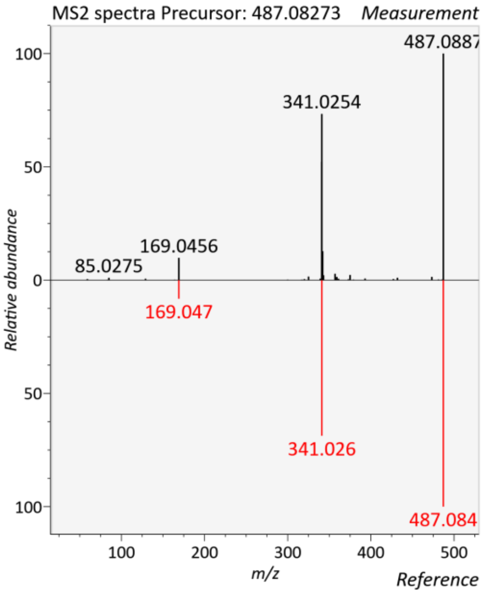

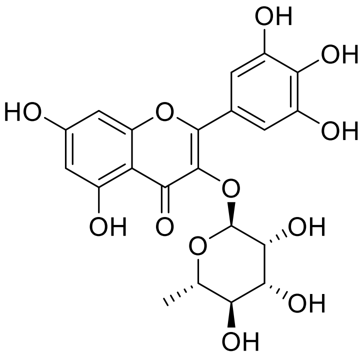

CID 5281673
Myricitrin
5054

Total Score 94.9

B. Myricetin (CID 5281672)

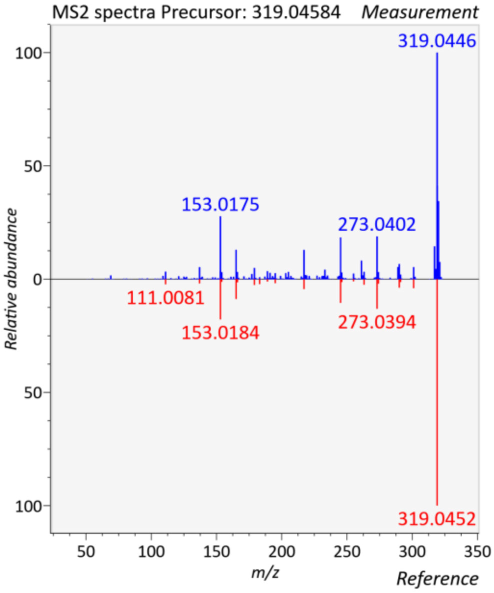

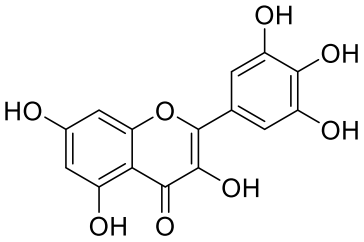

CID 5281672
Myricetin
2138

Total Score 88.4

C. Myricetin-3-O-pentoside (CID 21477996)

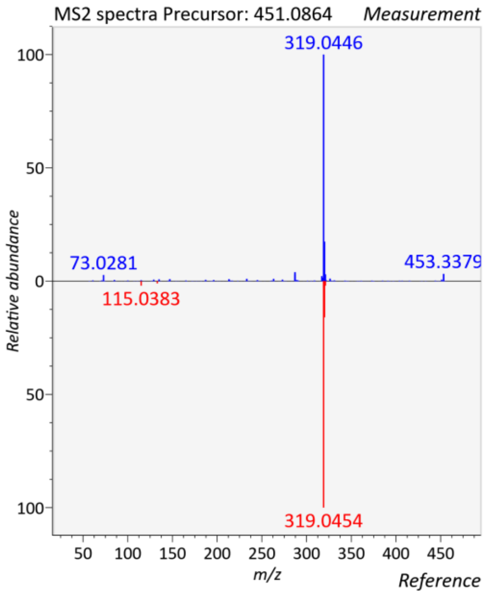

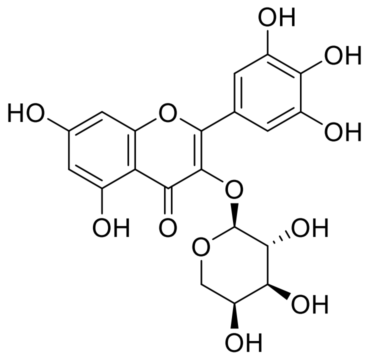

CID 21477996
Myricetin-3-O-pentoside
4761

Total Score 88.9

D. Myricetin-3-rutinside (CID 73803273)

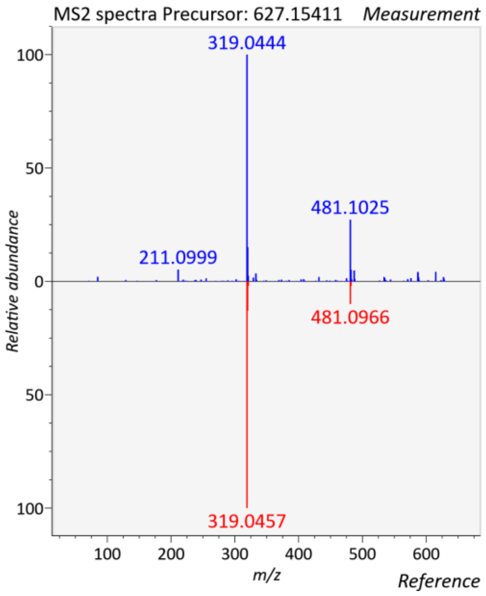

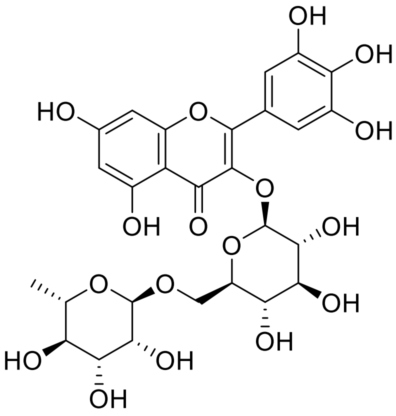

CID 73803273
Myricetin-3-rutinside
7819

Total Score 83.8

Figure S3. ^{1}H (400 MHz) and ^{13}C (100 MHz) NMR spectra

A. Myricitrin

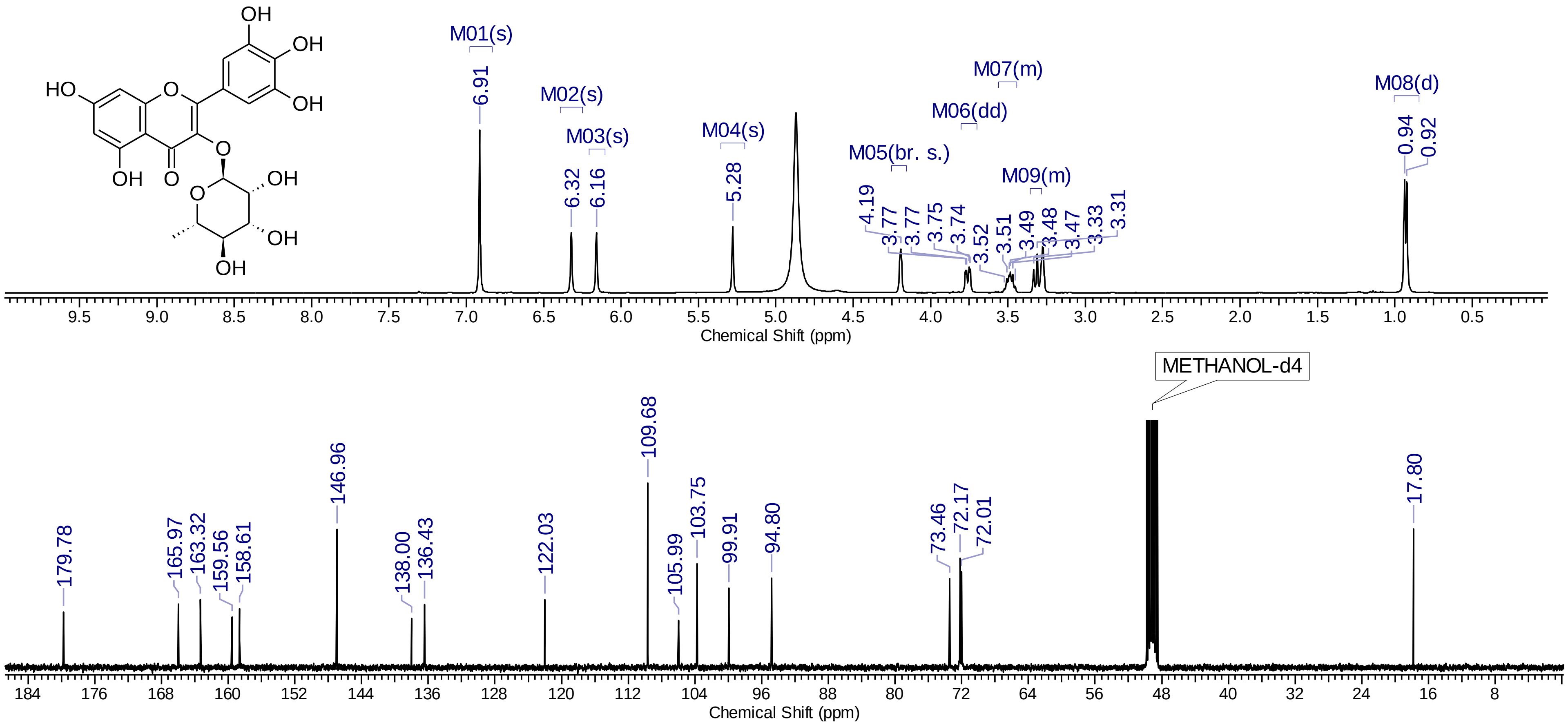

B. Myricetin

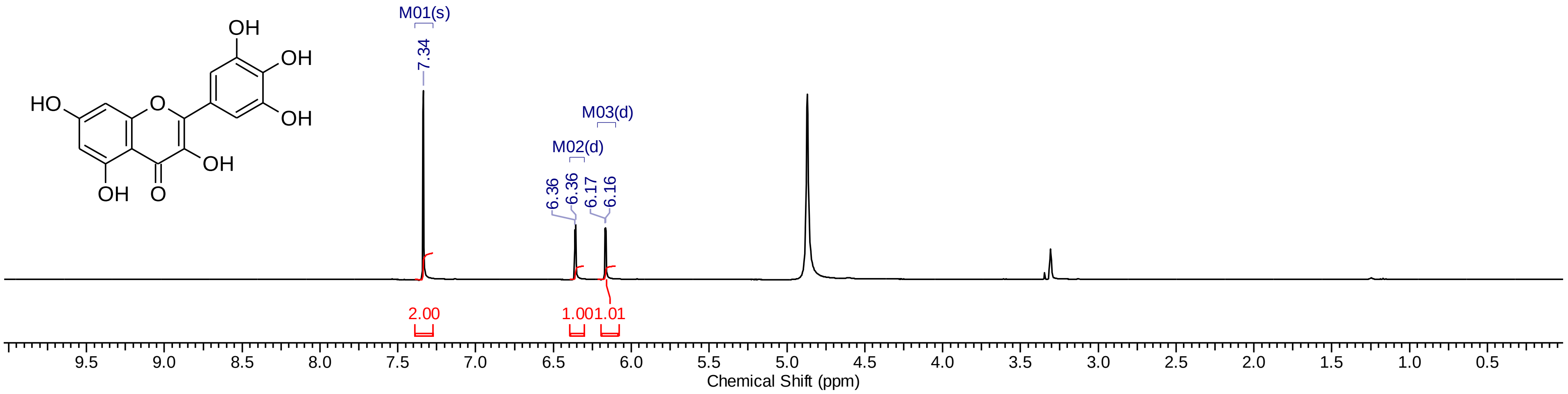
OH
OH
HO
O
OH
OH
OH
O
M01(s)
7.34
M02(d)
M03(d)
6.36
6.36
6.17
6.16
2.00
1.00
1.01
9.5
9.0
8.5
8.0
7.5
7.0
6.5
6.0
5.5
5.0
4.5
4.0
3.5
3.0
2.5
2.0
1.5
1.0
0.5
Chemical Shift (ppm)

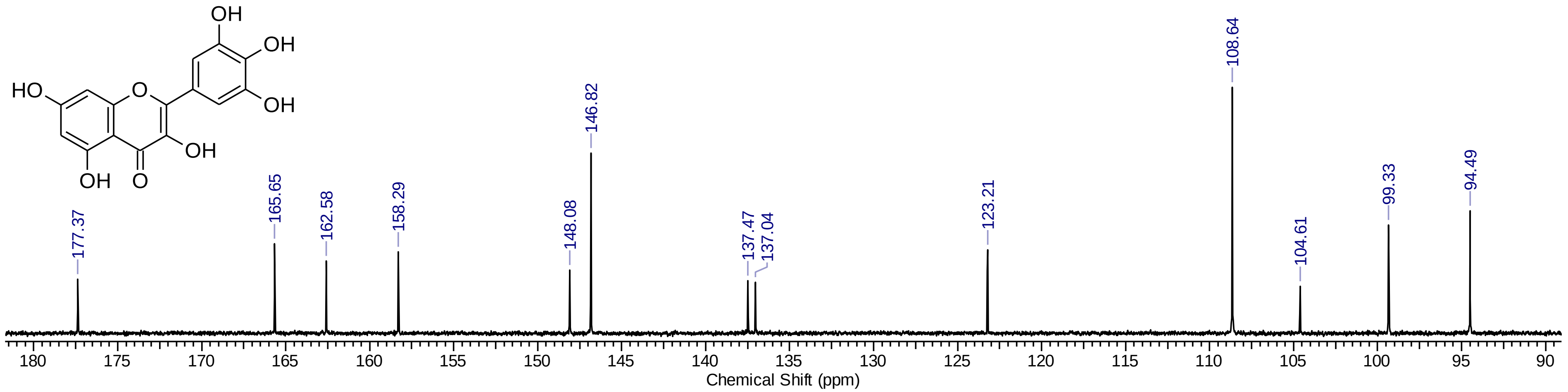
OH
OH
HO
O
OH
OH
OH
O
177.37
165.65
162.58
158.29
148.08
146.82
137.47
137.04
123.21
108.64
104.61
99.33
94.49
180
175
170
165
160
155
150
145
140
135
130
125
120
115
110
105
100
95
90
Chemical Shift (ppm)
